# Impact of liposomal delivery on coenzyme Q10 absorption: a double-blind, placebo-controlled, randomized trial

**DOI:** 10.3389/fnut.2025.1605033

**Published:** 2025-09-17

**Authors:** Ralf Jäger, Martin Purpura, Ashok Godavarthi, Halil Ibrahim Ceylan, Sebastian T. Balcombe, Ambrish Chandrappa, Grant M. Tinsley

**Affiliations:** ^1^Increnovo LLC, Whitefish Bay, WI, United States; ^2^Radiant Research Services Pvt. Ltd., Bangalore, India; ^3^Physical Education and Sports Teaching Department, Faculty of Sports Science, Atatürk University, Erzurum, Türkiye; ^4^Specnova LLC, Tysons Corner, VA, United States; ^5^Medstar Specialty Hospital, Bengaluru, India; ^6^Energy Balance & Body Composition Laboratory, Department of Kinesiology & Sport Management, Texas Tech University, Lubbock, TX, United States

**Keywords:** liposomes, coenzyme Q10, bioavailability, absorption, ubiquinone, pharmacokinetics, dietary supplements

## Abstract

**Background:**

Coenzyme Q10 (CoQ-10) plays a vital role in cellular energy production and protection against oxidative stress. However, its absorption from orally administered forms is limited due to its poor water solubility and relatively large molecular weight. While co-ingesting CoQ-10 with a fatty meal can enhance absorption, this approach is not always practical. The aim of this study was to evaluate whether a liposomal formulation of CoQ-10 could improve its absorption compared with standard CoQ-10 without the need for the concurrent consumption of fatty foods.

**Methods:**

In a randomized, double-blind, placebo-controlled, crossover study design, 7 men and 11 women (*n* = 18; age: 33.5 ± 6.4 years, height: 171.2 ± 8.1 cm, weight: 65.6 ± 8.8 kg) ingested a single dose of placebo (PLA), 100 mg of unformulated CoQ-10, or 100 mg of liposomal CoQ-10 (Lipo CoQ-10, LipoVantage®, Specnova, LLC, Tyson Corner, VA, USA). Venous blood samples were collected at 0, 0.5, 1, 1.5, 2, 3, 4, 6, 8, 12, and 24 h after ingestion and analyzed for plasma CoQ-10 concentrations.

**Results:**

CoQ-10and Lipo CoQ-10 demonstrated significantly greater Cmax and AUC0-24 compared with placebo (*p* < 0.001). Additionally, Lipo CoQ-10 had significantly higher Cmax (+31.3%, *p* < 0.001) and AUC0-24 (+22.6%, *p* < 0.001) values as compared with CoQ-10. CoQ-10 formulations were well-tolerated, with no significant changes in safety markers (blood pressure, renal function, liver enzymes, and lipid profile; *p* > 0.05), indicating a favorable safety profile.

**Conclusion:**

Liposomal delivery significantly enhances CoQ-10 absorption.

**Clinical trial registration:**

https://www.ctri.nic.in identifier CTRI/2024/04/066483.

## 1 Introduction

Coenzyme Q10 (2,3-dimethoxy-5-methyl-6-decaprenyl-1,4-benzoquinone, also known as ubiquinone, CoQ-10) is a vitamin-like, lipophilic molecule (1). It consists of a p-benzoquinone ring attached to a polyisoprenoid side chain, which is integral to its role as an electron and proton transporter within the mitochondria (2). CoQ-10 is involved in aerobic cellular respiration, facilitating the generation of energy in the form of ATP. CoQ-10 is found in nearly all eukaryotic cells, predominantly associated with the inner mitochondrial membrane. Beyond its role as an electron carrier, CoQ-10 functions as an antioxidant, protecting cellular components from oxidative damage caused by free radicals and stabilizing cell membranes (3).

CoQ-10 is present in all human and animal tissues, with concentrations varying depending on the type of tissue. Tissues with high energy demands or metabolic activity, such as the heart, kidneys, liver, and muscles, contain relatively high levels of CoQ-10 (1). As a lipophilic molecule, the distribution of CoQ-10 in tissues is influenced not only by its metabolic activity but also by the lipid content of the tissues. The absorption of CoQ-10 in the gastrointestinal tract follows a process similar to that of lipids. Its uptake mechanism resembles that of vitamin E, another lipid-soluble nutrient. CoQ-10 absorption is enhanced in the presence of lipids, and the absorption of supplemental CoQ-10 is further improved when ingested with a fatty meal (1). Similar to vitamin E and other lipophilic substances, CoQ-10 is incorporated into chylomicrons after absorption and transported through the lymphatic system into the circulation. The absorption efficiency of orally administered CoQ-10 is generally low due to its poor water solubility, limited solubility in lipids, and relatively large molecular size (4).

Due to its potential health benefits, CoQ-10 supplementation has garnered attention for a variety of applications, including cardiovascular health, neurodegenerative conditions, and aging. Despite its widespread commercial use, the bioavailability of CoQ-10 remains an area of active investigation. The absorption of CoQ-10 is highly dependent on its solubility in the gastrointestinal tract, which is often limited by its hydrophobic nature. Traditional CoQ-10 formulations, typically in crystalline or powder form, exhibit poor water solubility, leading to suboptimal absorption and inconsistent health outcomes (5). Recent advancements in nutrient delivery technologies, such as nanoemulsions, liposomes, and solubilized formulations, could be used to enhance CoQ-10 absorption and bioavailability (6). Liposomal delivery technology, which became commercially successful in the late 1990s, has been shown to improve the bioavailability and absorption of both hydrophilic and hydrophobic compounds compared to other oral forms of bioactive ingredients. This improvement is largely due to the protection offered against the harsh environment of the gastrointestinal tract and the enhanced transmucosal uptake and absorption (7). However, the impact of these novel formulations on CoQ-10 pharmacokinetics remains insufficiently understood. In the present study, the liposomal formulation utilizes a sunflower lecithin-based phospholipid bilayer with a proprietary ratio of phospholipids, combined with gum arabic and alginate. These polysaccharides form the polar core of the liposome and are also present externally, which may enhance gastrointestinal stability, protect against enzymatic degradation, and improve solubility and transmucosal absorption of CoQ-10. The inclusion of these specific excipients distinguishes this formulation from standard CoQ-10 formulations and provides the rationale for its direct comparison in this trial. Recently, the successful incorporation of vitamin C into this specific liposomal delivery system was shown to enhance its absorption into plasma and leukocytes (8). The leading hypothesis of this study was that a sunflower lecithin-based liposomal formulation containing gum arabic and alginate would significantly enhance CoQ-10 absorption compared with a standard formulation, even in a fasted state. The novelty of our work lies in the use of this specific proprietary composition and the application of a randomized, placebo-controlled, crossover design allowing direct pharmacokinetic comparison under identical conditions.

## 2 Methods

This study was a randomized, double-blind, placebo-controlled, parallel-group clinical trial designed to compare the pharmacokinetics, safety, and tolerability of a liposomal CoQ-10 formulation with placebo in healthy adults. The study was approved by the Medstar Specialty Hospital Ethics Committee on 20 March 2024 (IRB approval number: RRS/CL/BA/CoQ1/2024) and was registered with the Clinical Trials Registry, India (CTRI/2024/04/066483). The research was conducted at Medstar Specialty Hospital (Bengaluru, Karnataka 560092, India) and adhered to the principles set forth in the Declaration of Helsinki (Edinburgh, 2000) and the ICH-harmonized tripartite guidelines for Good Clinical Practice (GCP).

### 2.1 Participants

Twenty-two subjects were screened for this study. The sample size was determined based on previous studies of liposomal vitamin C (8). Subjects eligible for the study were required to meet the following criteria: female (non-pregnant) or male; aged 18–45 years; weighing at least 50 kg; in good health, with no evidence of underlying disease, as determined by medical history, physical examination, ECG, chest X-ray (PA view), and laboratory tests performed within 7 days prior to study commencement; and screening laboratory values within normal limits or considered by the Principal Investigator to be of no clinical significance. Subjects were excluded from the study if they met any of the following conditions: allergies to CoQ-10 products, food, or any other drugs; use of fat-reducing drugs, statins, or vitamin supplements (including vitamin E) within the past month; resting hypotension (BP < 90/60 mmHg), hypertension (BP > 140/90 mmHg), or abnormal pulse rate (below 50/min or above 100/min); a history or current presence of significant cardiovascular, pulmonary, hepatic, renal, hematological, gastrointestinal, endocrine, immunologic, dermatologic, neurological, musculoskeletal, or psychiatric conditions, or hospitalization/surgery within the past 4 weeks; a history of myocardial infarction (MI), stroke, peripheral arterial disease, gastrointestinal bleeding, hepatic impairment, asthma, renal impairment, epilepsy, or intracranial hemorrhage; use of over-the-counter or prescribed medications, including any enzyme-modifying drugs, within the past 14 days; history of alcoholism, drug abuse, or smoking; hypersensitivity to heparin; participation in any other clinical study within the past 3 months; and difficulty with blood donation, swallowing, or repeated venipuncture, or the presence of unsuitable veins for venipuncture. During the initial screening visit, subjects underwent several diagnostic assessments, including an electrocardiogram (ECG), chest X-ray (PA view), and hematological tests (red blood cell (RBC) count, hemoglobin, total and differential leukocyte count, and platelet count). Serum chemistry tests were performed, including a random blood sugar test (RBS), renal function tests (RFT)—creatinine and urea, and liver function tests (LFT)—total bilirubin, serum glutamic pyruvic transaminase (SGPT), and serum glutamic-oxaloacetic transaminase (SGOT). Serological tests included an HIV test and screening for Hepatitis B surface antigen. Urine analysis comprised physical examination (color, appearance, and specific gravity), chemical examination (pH, protein, glucose, bile salt, and bile pigments), and microscopic examination (pus cells, epithelial cells, bacteria, RBCs, casts, and crystals). At the time of study design, no published liposomal CoQ-10 pharmacokinetic trials with comparable methodology (fasted state, crossover design, and identical dose) were available to conduct a sample size estimation. Therefore, we used variability estimates from our prior liposomal vitamin C study (8), conducted with the same delivery platform, to ensure methodological relevance.

### 2.2 Study procedure

This study was conducted as a randomized, double-blind, placebo-controlled, and crossover trial. Initially, 22 participants were assessed for eligibility; however, four participants were excluded due to smoking (*n* = 2), alcohol consumption (*n* = 1), and difficulty in donating blood (*n* = 1). The remaining 18 eligible participants were randomly assigned to three groups, each consisting of six participants, using simple randomization. To preserve the blinding of the study treatments, all test products were identical in appearance. The study materials were coded centrally, with randomization numbers assigned according to a computer-generated randomization schedule.

**Figure 1:**
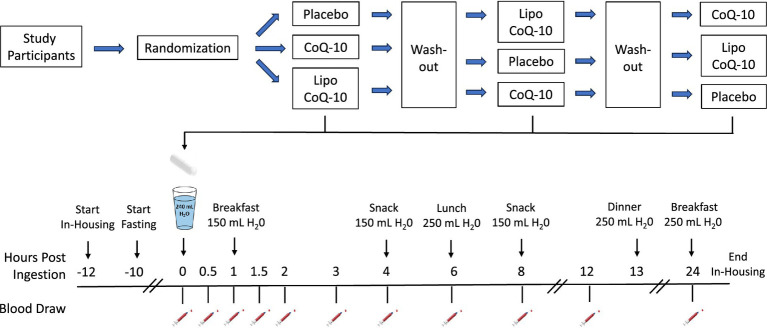
Schematic overview of research design.

Subjects who met the inclusion and exclusion criteria were checked into the clinical facility at least 12 h prior to the administration of the test products. During each visit, subjects were seated comfortably, and a qualified phlebotomist inserted a catheter into a forearm vein. A baseline blood sample was obtained, and one of three treatment dosages (CoQ-10, Lipo CoQ-10, or PLA) was administered with 240 mL of water at ambient temperature. Blood samples were collected at 0.5, 1, 1.5, 2, 3, 4, 6, 8, 12, and 24-h intervals postadministration of the test product. Each subsequent trial was separated by a minimum of 3 days for washout and followed identical procedures, except for the formulation of CoQ-10 or placebo consumed (see Figure 1). During the study period, subjects were prescribed a CoQ-10-free diet, with meals consisting of 200 mL of apple juice, two bread rolls, and 15 g of butter. Subjects remained in the clinical facility for at least 24 h postdose.

### 2.3 Study materials

CoQ-10 capsules, liposomal CoQ-10 capsules (Lipo CoQ-10, LipoVantage^®^, Specnova, LLC, Tysons Corner, VA, USA), and placebo capsules (PLA, maltodextrin) were obtained from Molecules Food Solutions Pvt. Ltd., Kerala, India. Subjects ingested one optically identical hard gel capsule of each study material per session, with each capsule providing 100 mg of CoQ-10, or a placebo. The CoQ-10 content was verified through independent third-party analysis (Interfield Laboratories, Kochi, India). The liposomal formulation of CoQ-10 contained coenzyme Q10, sunflower lecithin (with a proprietary ratio of phospholipids), gum arabic, and alginate. These polysaccharides constitute the polar core of the liposome and are also present on the exterior. The liposomal structure was confirmed using transmission electron cryomicroscopy (CryoTEM) with a Thermo Fisher Scientific Titan Krios G4 cryo-transmission electron microscope (300 kV), equipped with a Falcon 4 direct electron detector, and a Thermo Fisher Scientific Talos F200i (S)TEM (20–200 kV field-emission scanning/transmission electron microscope).

### 2.4 Safety

Safety was evaluated by monitoring vital signs throughout the study, assessing the frequency and severity of adverse events, and analyzing changes in blood parameters. Blood parameters included hemoglobin, total leukocyte count, RBC count, platelet count, neutrophils, lymphocytes, eosinophils, monocytes, basophils, erythrocyte sedimentation rate (ESR), blood urea nitrogen (BUN), blood uric acid, serum creatinine, serum bilirubin, serum glutamic-oxaloacetic transaminase (SGOT), serum glutamic pyruvic transaminase (SGPT), serum alkaline phosphatase, serum albumin, serum sodium, serum potassium, serum chloride, blood glucose, and lipid profile (total cholesterol, triglycerides, high-density lipoprotein (HDL), low-density lipoprotein (LDL), very low-density lipoprotein (VLDL)). Blood samples were analyzed by Radiant Research Services Private Limited, # 99/A, 8 Main, III Phase, Peenya Industrial Area, Bangalore, Karnataka, 560,058, India. Adverse events and changes in blood parameters were compared from baseline to the final visit. Vital signs, including blood pressure (measured in a sitting or semi-supine position), radial pulse rate, and oral temperature, were assessed at several time points: admission, pre-dosing (0 h), 15 min, 30 min, 60 min, 120 min, 4 h, 8 h, 12 h postdose, and at checkout (24 h postdose). A window of ± 15 min was allowed for postdose vital sign recordings. Physical examinations were conducted at admission and at 1-, 2-, 4-, 8-, 12-, and 24-h postdose (see Table 1 for schedule of events). Although CoQ-10 at a dosage of 100 mg is widely recognized as safe and well-tolerated, safety parameters were included in the present trial in accordance with Good Clinical Practice and local ethics committee requirements. This ensured comprehensive clinical monitoring and confirmed that the liposomal delivery system did not introduce any unexpected adverse effects.

**Table 1:** Schedule of events.

Procedure	Timeline								
	Screening visit	Period I			Period II			Period III	
		Visit 1	Visit 2		Visit 1	Visit 2		Visit 1	Visit 2
	Day-7	Day 0	Day 1		Day 0	Day 1		Day 0	Day 1
Informed consent	X	–	–	Wash out	–	–	Wash out	–	–
Demographics									
Gender, age, height, body weight	X	X	X		X	X		X	X
Medical history	X	–	–		–	–		–	–
Eligibility assessment and randomization	–	X	–		X	–		X	–
Concomitant medication review	X	X	X		X	X		X	X
Physical examination	X	X	–		X	–		X	–
ECG	X	–	–		–	–		–	–
Chest X-ray	X	–	–		–	–		–	–
Safety parameters									
Basic hematology, biochemistry, and urine	X	X	X		X	X		X	X
Serology									
HIV, Hepatitis B surface antigen	X	–	–		–	–		–	–
Biomarkers									
CRP, MDA, GPx-1, TNF-𝛼	–	X	X		X	X		X	X
IP administration	–	X	–		X	–		X	–
Blood sample collection at specified time points		X	X		X	X		X	X
Vital signs									
Temperature, pulse, BP	–	X	X		X	X		X	X

ECG, electrocardiogram; HIV, Human Immunodeficiency Virus; CRP, C-Reactive Protein; MDA, Malondialdehyde; TNF-𝛼, Tumor Necrosis Factor-Alpha; GPx-1, Glutathione Peroxidase 1, BP, Blood Pressure.

### 2.5 Sample collection

Biochemical parameters were analyzed by Radiant Research Services Pvt. Ltd. (Bangalore, India) using standard automated clinical chemistry analyzers and hematology systems, following manufacturer instructions and internal quality control procedures. At each time point throughout the study, 10 mL of blood was drawn from the catheter into vacutainer tubes. An additional 11 mL of blood was collected during the screening visit and 24 h postdose for safety parameter analysis. Blood draws were collected within ± 6 min of the scheduled times. On the day of the study, phlebotomy was performed within 1 h prior to dosing. An indwelling IV cannula or scalp vein was placed in a forearm vein of the subjects, which remained in place until the 12-h mark. Blood samples were collected using prechilled, labeled EDTA plastic tubes and immediately placed in a wet ice bath until centrifugation. After collection, all blood samples were kept in a wet ice bath. Once samples from all participants at each time point were obtained, they were processed by centrifugation at 5,000 ± 100 RPM for 15 min. Plasma samples were transferred into appropriately labeled microcentrifuge tubes using a micropipette and stored in a deep freezer at −20°C for interim storage. Every 2 h, the collected plasma samples were transferred to a − 80°C deep freezer for long-term storage. Upon completion of the study, all samples were transported to the analytical laboratory for analysis.

### 2.6 Sample preparation and analysis

Blank plasma samples were prepared for Liquid chromatography-tandem mass spectrometry (LC-MS/MS) analysis using the protein precipitation (PPT) method. Prior to analysis, the samples were removed from the deep freezer and allowed to thaw at room temperature. To prepare the plasma samples, 50 μL of plasma was transferred to a 2.0 mL centrifuge tube, and 150 μL of the precipitating agent (0.1% formic acid in acetonitrile) was added. The mixture was then vortexed for 2 min. The resulting solution was centrifuged at 4,000 rpm for 7 min. The supernatant was carefully separated and injected into the LC–MS/MS system for analysis.

The LC–MS/MS conditions for CoQ-10 analysis were as follows: The LC system used was LC AC, and the mass spectrometer was a SCIEX 4000. The ion source was operated in electrospray ionization (ESI) mode. A Kinetex Biphenyl column (100 × 4.6 mm, 3 μm) was employed for chromatographic separation. The collision gas pressure was set to 12 psi, while the curtain gas was maintained at 30 psi and the ion source gas at 55 psi. The ion spray voltage was set to 5,500 V and the source temperature was maintained at 450°C. The column oven temperature was set to 40°C. The mobile phase A consisted of acetonitrile, 2-propanol, and formic acid in a ratio of 90:10:0.1%. A 20 μL injection volume was used for each sample. The ions monitored were M—863.7 > 197.1. The analysis was conducted in isocratic mode with a flow rate of 0.6 mL/min for a total run time of 5.0 min. The coefficient of determination for the calibration curve was R^2^ = 0.99. The limit of quantification (LOQ) was 25.0 ng/mL, while the limit of detection (LOD) was 10.0 ng/mL.

### 2.7 Statistical analysis

Statistical analyses were planned to compare treatment groups for primary and secondary endpoints using methods appropriate for normally and non-normally distributed data, ensuring robust evaluation of both pharmacokinetic and safety outcomes. Outcomes of interest included pharmacokinetic variables (Cmax, AUC0-24, Tmax) and percent changes from baseline (0 h) to 24 h after supplement ingestion in selected biomarkers (CRP, MDA, GPx-1, and TNF-𝛼) and safety indicators. To account for outliers and violations of the normal distribution, data were analyzed using the non-parametric Friedman test, with treatment (Liposomal CoQ-10, non-liposomal CoQ-10, and placebo) specified as a within-subjects factor. Following a statistically significant effect of treatment, *post hoc* Wilcoxon signed-rank tests were performed with the Bonferroni correction for multiple comparisons. For all tests, statistical significance was accepted at *p* < 0.05. Descriptive data are presented as median ± interquartile range (IQR) unless otherwise noted. Percent differences were calculated by dividing the absolute difference between values by their average and then multiplying by 100 to express the result as a percentage. Data were analyzed in R software v. 4.4.0 (9) with the rstatix package v. 0.7.2 (10).

## 3 Results

### 3.1 Participant characteristics

**Figure 2:**
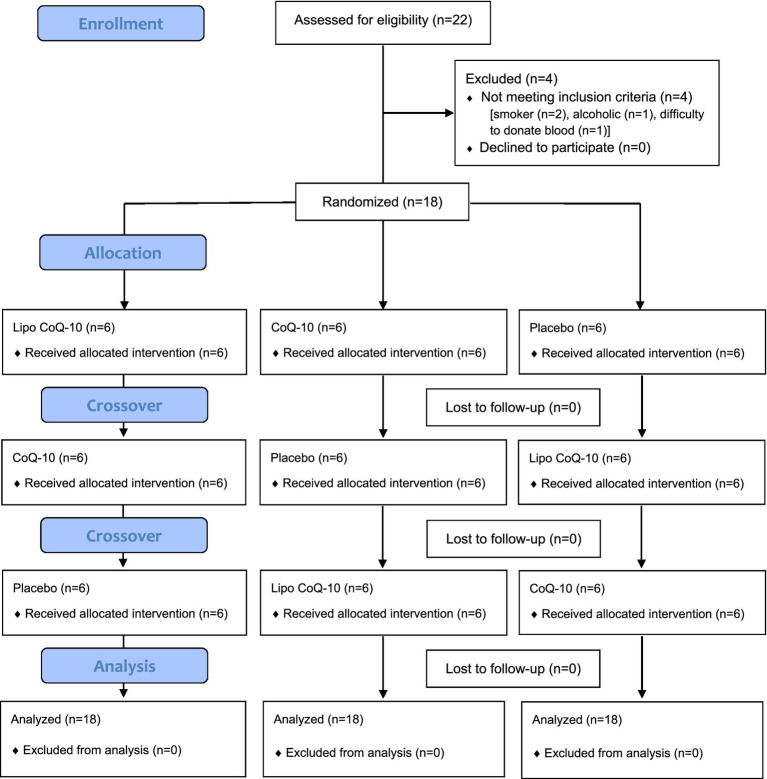
CONSORT flow diagram.

Eighteen participants (*n* = 7 M, *n* = 11 F) completed the present study and were included in the analysis (Table 2; Figure 2).

**Table 2:** Participant characteristics.

Characteristics	All (*n* = 18)		Males (*n* = 7)		Females (*n* = 11)	
	Mean	SD	Mean	SD	Mean	SD
Age (y)	33.5	6.4	35.3	8.3	32.4	4.9
Height (cm)	171.2	8.1	176.6	5.4	167.7	7.8
Body weight (kg)	65.6	8.8	74.3	5.0	60.1	5.4
BMI (kg/m^2^)	22.4	2.4	23.9	2.8	21.4	1.4
SBP (mmHg)	110.6	8.0	111.4	6.9	110.0	8.9
DBP (mmHg)	75.4	9.0	77.1	9.5	74.4	9.0

BMI, Body Mass Index; SBP, Systolic Blood Pressure; DBP, Diastolic Blood Pressure.

### 3.2 Pharmacokinetics analysis

**Figure 3:**
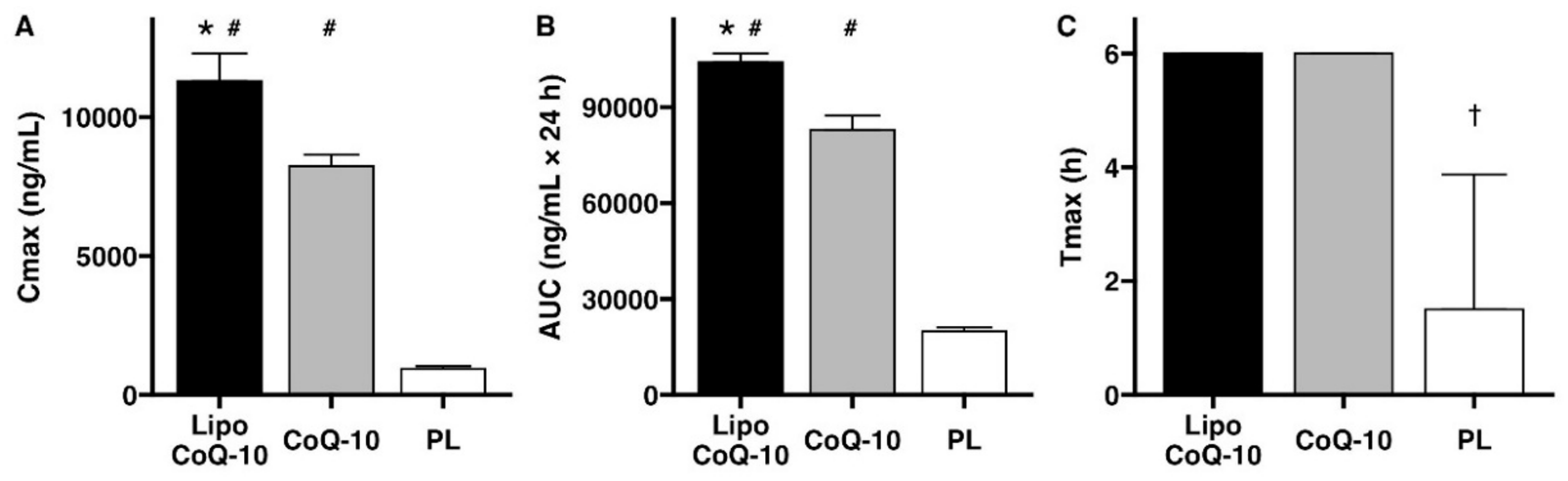
Pharmacokinetic comparison of Coenzyme Q-10 absorption. A significant benefit of liposomal delivery for maximal concentrations (Cmax, **A**) and area under the curve over 24 hours (AUC0-24, **B**) was observed, with no differences for time to maximum concentration (Tmax, **C**). #Indicates significant difference compared to placebo (PLA). *Indicates significant difference compared to standard Coenzyme Q-10. ^†^indicates significant difference compared to both liposomal and standard Coenzyme Q-10. Bars indicate median values ± IQR.

**Figure 4:**
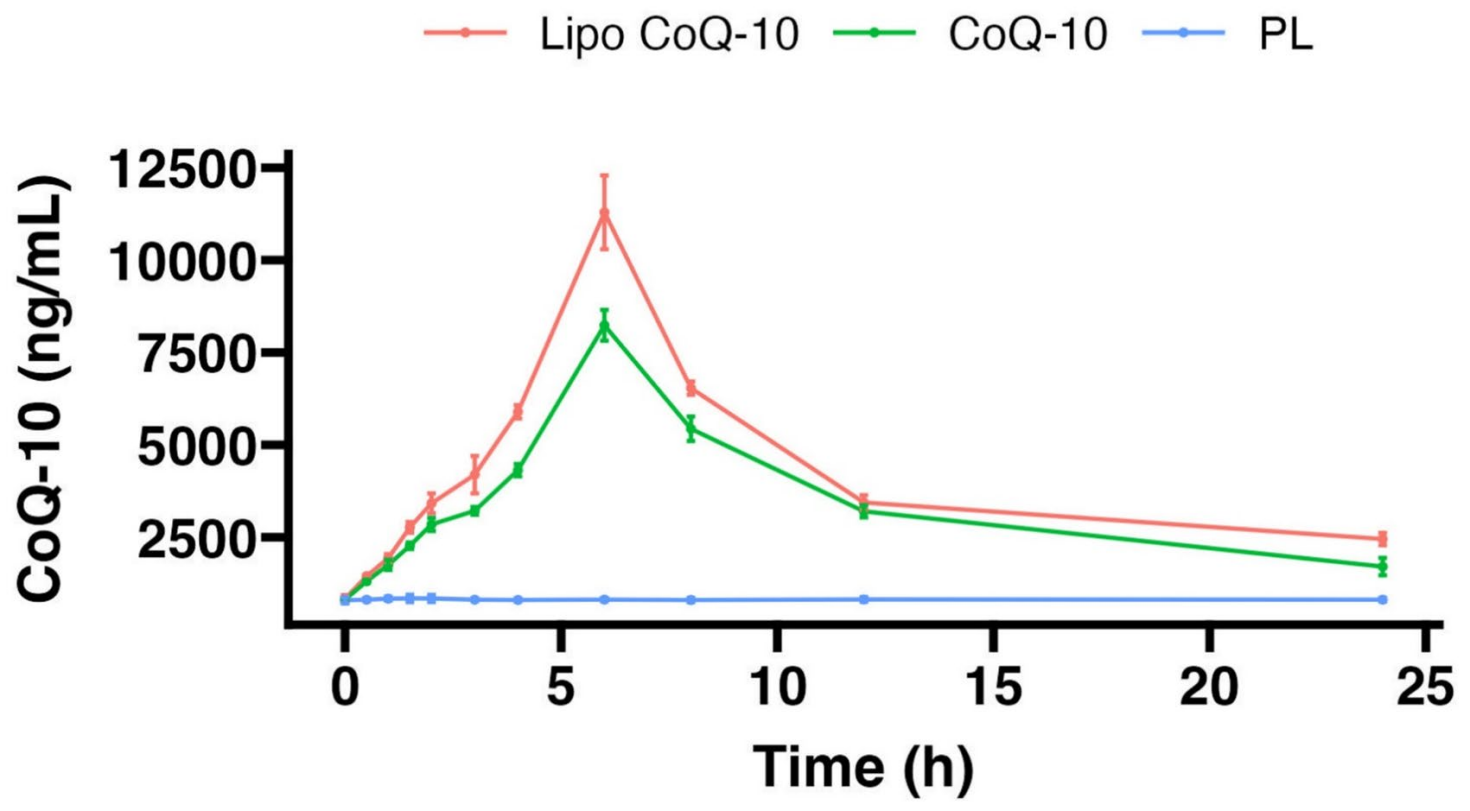
Effects of liposomal delivery on coenzyme Q-10 absorption. Raw concentrations of coenzyme Q-10 are displayed for 24 h following ingestion of liposomal coenzyme Q-10 (Lipo CoQ-10), standard coenzyme Q-10 (CoQ-10), or placebo (PL). Points indicate median values, and error bars indicate IQR. Cmax and AUC significantly differed between conditions, with higher values in Liposomal CoQ-10 as compared to non-liposomal CoQ-10 and placebo (*p* < 0.001 for each comparison).

Cmax significantly differed between conditions (Liposomal CoQ-10: 11,294 ± 998 ng/mL, Non-liposomal CoQ-10: 8,236 ± 417 ng/mL, Placebo 930 ± 100 ng/mL; *p* < 0.001; Figure 3A). Additionally, each condition significantly differed based on *post hoc* tests (*p* < 0.001 for each comparison). The median Cmax with Liposomal CoQ-10 was 31.3% higher than that of non-liposomal CoQ-10 and 169.6% higher than that of placebo, as measured by percent difference. Additionally, the median Cmax with non-liposomal CoQ-10 was 159.4% higher than that of placebo. Raw concentrations of CoQ-10 are displayed in Figure 4.

AUC0-24 significantly differed between conditions (Liposomal CoQ-10: 104,051 ± 2,762 ng/mL × 24 h, Non-liposomal CoQ-10: 82,934 ± 4,413 ng/mL × 24 h, Placebo 19,851 ± 1,316 ng/mL × 24 h; *p* < 0.001; Figure 3B). Additionally, each condition significantly differed based on *post hoc* tests (*p* < 0.001 for each comparison). The median AUC0-24 with Liposomal CoQ-10 was 22.6% higher than that of non-liposomal CoQ-10 and 135.9% higher than that of placebo, as measured by percent difference. Additionally, the median AUC0-24 with non-liposomal CoQ-10 was 122.7% higher than that of the placebo.

For Tmax, all participants in the liposomal CoQ-10 and non-liposomal Co-Q10 conditions demonstrated a Tmax value of 6 h (Figure 3C). For PLA, Tmax values were 1.5 ± 2.4 h. A significant difference between conditions (*p* < 0.001) was observed, with follow-up testing confirming this finding was due to differences between PLA and each experimental group (*p* = 0.01 for each).

### 3.3 Biomarkers

**Figure 5:**
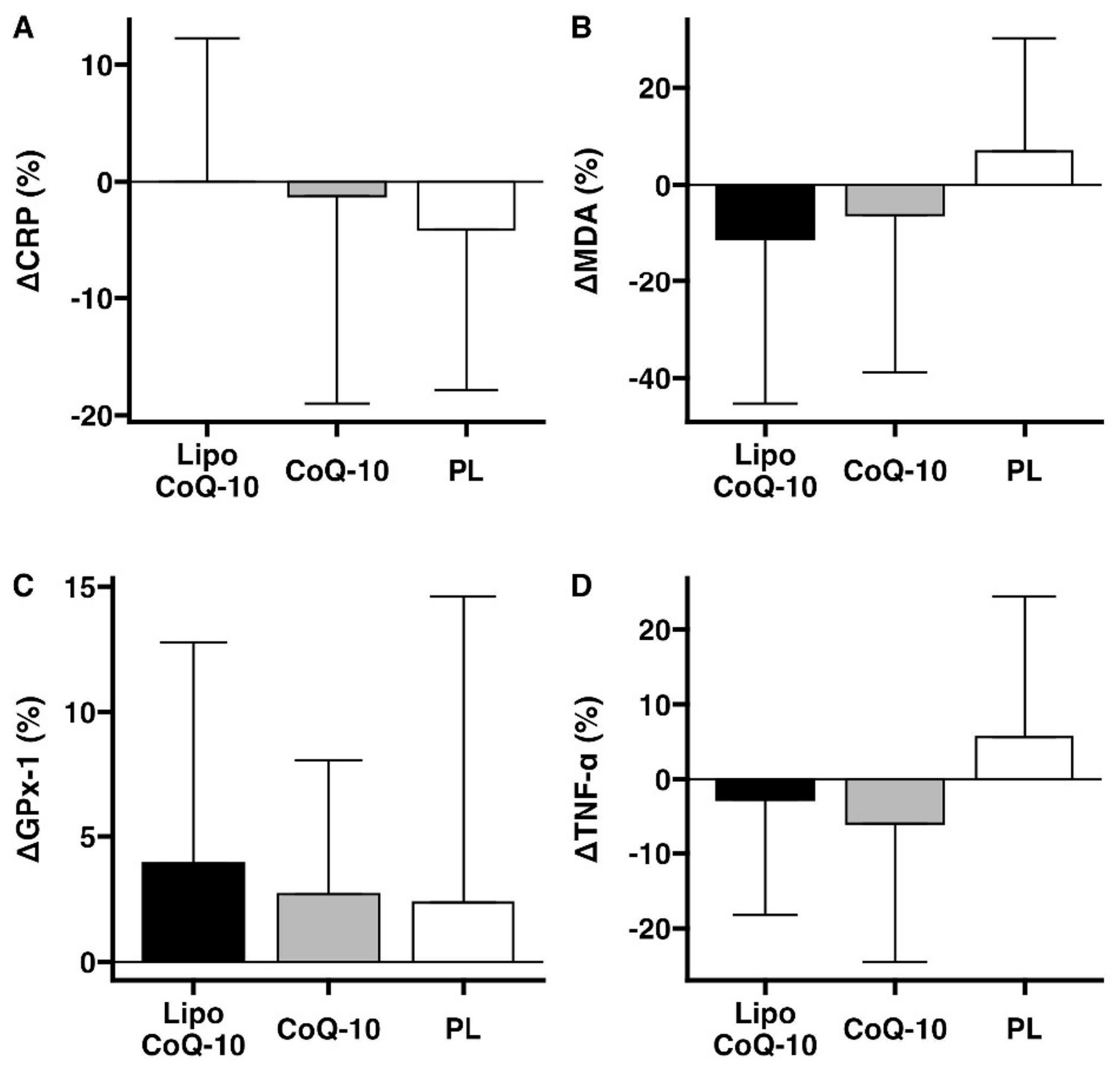
Biomarker changes after supplement ingestion. The potential effects of liposomal and standard delivery of coenzyme Q-10, as compared to placebo, were examined for C-reactive protein **(A)**, malondialdehyde **(B)**, glutathione peroxidase 1 **(C)**, and tumor necrosis factor alpha **(D)**. Bars indicate median percent changes ± IQR. Data are presented as mean ± SD. This exploratory analysis was conducted to assess whether the enhanced absorption observed with the liposomal formulation could result in measurable acute efficacy-related differences. Future studies with larger cohorts, repeated dosing, and increased sampling frequency are warranted to confirm these preliminary findings.

No significant differences between treatments were observed for changes in CRP over each 24-h treatment period (liposomal CoQ-10: 0.0 ± 12.2%, non-liposomal CoQ10: −1.2 ± 17.8%, placebo: −4.1 ± 13.8%; *p* = 0.61; Figure 5A). Similarly, no differences between treatments were observed for changes in MDA (liposomal CoQ-10: −11.1 ± 34.2%, non-liposomal CoQ10: −6.2 ± 32.6%, placebo: 6.9 ± 23.3%; *p* = 0.50; Figure 5B). GPx-1 changes did not differ between treatments (liposomal CoQ-10: 3.9 ± 8.8%, non-liposomal CoQ10: 2.7 ± 5.4%, placebo: 2.4 ± 12.2%; *p* = 0.94; Figure 5C). Finally, no differences between treatments were observed for TNF-𝛼 (liposomal CoQ-10: −2.7 ± 15.5%, non-liposomal CoQ-10: −6.1 ± 18.4%, placebo: 5.6 ± 18.7%; *p* = 0.31; Figure 5D).

### 3.4 Safety markers

The supplements were well tolerated, and none of the subjects reported any adverse events during the ingestion of any of the three study treatments. No statistically significant effects of treatment were observed for changes in multiple safety markers, including blood pressure, urinary markers, and blood makers (Table 3).

**Table 3:** Percent changes in safety markers from baseline to 24 h after supplement ingestion (*p* values from Friedman tests).

Variable	Treatment	Median (%)	IQR (%)	*p*
SBP (mmHg)	Lipo CoQ-10	−0.5	3.6	0.34
	CoQ-10	−2.5	8.7	
	PL	1.9	4.8	
DBP (mmHg)	Lipo CoQ-10	0.0	7.3	0.05
	CoQ-10	0.0	2.8	
	PL	2.7	7.4	
Urea (mg/dL)	Lipo CoQ-10	−4.6	5.0	0.50
	CoQ-10	−3.9	15.3	
	PL	−6.6	16.4	
Creatinine (mg/dL)	Lipo CoQ-10	0.0	10.7	0.96
	CoQ-10	0.0	13.4	
	PL	0.0	23.4	
Uric Acid (mg/dL)	Lipo CoQ-10	0.0	2.0	0.41
	CoQ-10	0.0	3.6	
	PL	0.0	5.3	
RBS (mg/dL)	Lipo CoQ-10	−4.9	8.4	0.68
	CoQ-10	−3.9	2.8	
	PL	−4.1	4.3	
SGOT (U/L)	Lipo CoQ-10	−5.7	7.9	0.55
	CoQ-10	−3.8	9.0	
	PL	−7.6	5.6	
SGPT (U/L)	Lipo CoQ-10	−6.6	13.7	0.26
	CoQ-10	−1.9	11.4	
	PL	−3.2	7.3	
Bilirubin (mg/dL)	Lipo CoQ-10	0.0	25.2	0.10
	CoQ-10	−11.8	25.0	
	PL	−4.5	22.9	
Alkaline phosphatase (U/L)	Lipo CoQ-10	−1.4	2.6	0.50
	CoQ-10	−1.2	4.9	
	PL	−1.3	5.1	
Albumin (g/dL)	Lipo CoQ-10	−3.6	3.0	0.30
	CoQ-10	−3.6	3.4	
	PL	−2.9	8.7	
Triglycerides (mg/dL)	Lipo CoQ-10	−4.8	3.1	0.83
	CoQ-10	−4.5	4.3	
	PL	−6.5	3.5	
HDL (mg/dL)	Lipo CoQ-10	−2.2	2.0	0.35
	CoQ-10	−1.8	1.5	
	PL	−2.1	1.3	
LDL (mg/dL)	Lipo CoQ-10	−1.6	1.9	0.65
	CoQ-10	−1.2	2.3	
	PL	−2.0	2.2	
VLDL (mg/dL)	Lipo CoQ-10	0.0	10.4	0.66
	CoQ-10	0.0	10.3	
	PL	4.8	5.6	

SBP, Systolic Blood Pressure; DBP, Diastolic Blood Pressure; RBS, Random Blood Sugar; SGOT, Serum Glutamic-Oxaloacetic Transaminase; SGPT, Serum Glutamic-Pyruvic Transaminase; HDL, High-Density Lipoprotein; LDL, Low-Density Lipoprotein; VLDL, Very Low-Density Lipoprotein; IQR, Interquartile Range; PL, Placebo.

## 4 Discussion

This study investigated the bioavailability of a liposomal formulation of CoQ-10 compared with standard CoQ-10. The results indicate that liposomal administration significantly enhances the absorption of CoQ-10, as shown by the increased peak plasma concentration (Cmax: 11,294 ± 998 ng/mL) and total exposure (AUC0-24: 104,051 ± 2,762 ng/mL × 24 h) associated with liposomal CoQ-10. The liposomal formulation of CoQ-10 had a 31.3% higher Cmax and a 22.6% higher AUC0-24 over 24 h compared with the standard formulation (*p* < 0.001), indicating a remarkable improvement in absorption efficiency. These results are consistent with previous research showing that liposomal encapsulation improves the bioavailability of nutrients by increasing solubility, protecting against degradation in the gastrointestinal tract, and facilitating absorption through biological membranes (7). A recent study using the same liposomal formulation showed significantly higher Cmax and AUC values for vitamin C compared to a non-liposomal formulation, highlighting the efficacy of liposomal administration in improving absorption (8).

The importance of formulation for the bioavailability of CoQ-10 was also demonstrated by López-Lluch et al. (11), who conducted a double-blind crossover study in 14 healthy individuals comparing seven different CoQ-10 supplement formulations. Their results showed significant differences in absorption, with area under the curve (AUC) values ranging from 2.45 to 25.15 mg L 48 h^−1^ over 48 h, depending on the formulation. Similarly, changes in Cmax ranged from 0.18 to 0.95 mg/L, highlighting the critical role of formulation in absorption efficiency. Soft gel capsules containing ubiquinone (oxidized CoQ-10) or ubiquinol (reduced CoQ-10) had the highest bioavailability, highlighting the importance of excipients, solubilization techniques, and individual physiological factors in the absorption of CoQ-10.

In addition, the CoQ-10 formulation used in the present study, consisting of sunflower lecithin, gum arabic, and alginate with a proprietary phospholipid ratio, plays a crucial role in improving stability, absorption, and bioavailability. This encapsulation method improves the solubility and stability of CoQ-10 in the gastrointestinal tract and enables better absorption compared with non-liposomal forms (5). Absorption of CoQ-10 is inherently complex (5) and exhibits considerable interindividual variability, which should be taken into account when evaluating different formulations (11, 12). This variability is influenced by differences in formulation type, delivery mechanism, and solubility enhancement strategies. For example, a comparative study investigating the absorption of a single dose and bioavailability of 100 mg CoQ-10 in three different lipid-based soft gels (crystal-free formulations), three crystalline CoQ-10 formulations, and three CoQ-10 dry powder formulations found that the crystal-free soft gel formulations showed better absorption over 24 h (13). Similarly, another study showed that the absorption of 60 mg CoQ-10 was enhanced by increasing its water solubility through complexation with *β*-cyclodextrin, resulting in improved absorption over 12 h (14). These results demonstrate the importance of optimizing CoQ-10 formulations to improve bioavailability and minimize absorption variability in different individuals.

Furthermore, the observation that both the liposomal and standard CoQ-10 formulations have a Tmax of 6 h is consistent with pharmacokinetic studies on the absorption of CoQ-10. In these studies, Tmax values of approximately 6 h were reported for various CoQ-10 formulations, which is consistent with the slow absorption profile attributed to the hydrophobicity and large molecular weight of CoQ-10 (15, 16). In contrast, the reported placebo Tmax of 1.5 ± 2.4 h is unexpected, as placebos typically do not exhibit measurable absorption kinetics. This discrepancy could be due to methodological factors, such as baseline CoQ-10 levels or analytical sensitivity that detects minor variations unrelated to supplementation. The lack of different absorption kinetics of liposomal CoQ-10 compared with standard forms in human studies is noteworthy despite theoretical expectations (17). In addition, higher doses of CoQ-10 show a dose-proportional increase in plasma concentration without significantly altering Tmax. Overall, these results underscore the need for further research to clarify placebo Tmax observations, particularly in studies that control dietary CoQ-10 intake and use standardized pharmacokinetic protocols.

In terms of biomarkers, a single dose of CoQ-10 is not known to significantly alter biomarkers of inflammation (CRP and TNF-𝛼) or oxidative stress (MDA and GPx-1). However, we speculated that if a liposomal formulation increased the absorption of CoQ-10, the mean changes in these biomarkers might be more significant in the liposomal group compared with the non-liposomal formulation, which would provide an early indication of potentially improved efficacy. However, this would need to be validated in a repeated-dose study. Interestingly, although the liposomal CoQ-10 had better pharmacokinetic properties, no significant differences were observed in these biomarkers compared with baseline. However, mean improvements were observed in oxidative stress markers, MDA (Lipo CoQ-10: −11.1 ± 34.2% vs. CoQ-10: −6.2 ± 32.6%), an indicator of lipid peroxidation, TNF-𝛼 (Lipo CoQ-10: −2.7 ± 15.5%, CoQ-10: −6.1 ± 18.4%) and GPx-1 (Lipo-CoQ-10: 3.9 ± 8.8% vs. CoQ-10: 2.7 ± 5.4%), with the reduced GPx-1 values indicating a possible increase in oxidative stress. This could be due to the short duration of the study. These results suggest that although liposomal CoQ-10 increases bioavailability, a single administration may not be sufficient to induce acute changes in systemic oxidative stress or inflammation. For example, a recent meta-analysis from 2023 confirmed that CoQ-10 supplementation reduces inflammatory markers, such as TNF-*α* and IL-6, with significant effects observed with interventions lasting more than 10 weeks (18). Previous studies have shown that the antioxidant and anti-inflammatory effects of CoQ10 supplementation are more pronounced with long-term use. Fan et al. (19) analyzed 17 randomized controlled trials in their systematic review and meta-analysis. They found that CoQ10 supplementation significantly reduced inflammatory markers, with weighted mean differences of −0.35 mg/L for CRP, −1.61 pg./mL for IL-6, and −0.49 pg./mL for TNF-α. However, these effects were more pronounced when supplementation lasted longer. Future studies with longer intervention periods are needed to investigate the potential effects of liposomal CoQ-10 on these biomarkers.

Regarding safety, the present study showed no significant changes in blood pressure, renal function markers, liver enzymes, or lipid profiles (*p* > 0.05). These results indicate that both the liposomal and standard CoQ-10 formulations are well tolerated and have no short-term adverse metabolic or cardiovascular effects in healthy individuals. These results align with prior evidence that CoQ-10 is safe and well-tolerated in humans. Hidaka et al. (20) reported an observed safety level of up to 1,200 mg/day based on multiple clinical trials. In a randomized controlled trial in patients with coronary heart disease receiving statin therapy, Lee et al. (21) found that 300 mg/day of CoQ-10 for 12 weeks showed no adverse effects on metabolic or cardiovascular markers, while it improved antioxidant enzyme activity and reduced inflammation. A meta-analysis also found that CoQ-10 supplementation modestly reduced SBP without affecting DBP in patients with metabolic diseases (22). Additional studies indicate potential benefits for liver health (23), lipid profiles (24), and possibly renal function, particularly when combined with selenium (25). Collectively, these findings, together with our results, support the safety of CoQ-10, including liposomal formulations, at higher doses, with possible added cardiometabolic benefits in at-risk populations.

This study has several strengths, including the controlled internal environment that minimized external influences such as environmental factors, sleep variability, and uncontrolled physical activity. The use of standardized meals eliminated variability in the diet, ensuring that differences in absorption were due to the recipe and not the composition of the food. In addition, the 24-h blood sampling protocol provided a comprehensive pharmacokinetic profile that captured Cmax, AUC0-24, and Tmax, which are essential parameters for assessing the bioavailability of CoQ-10. The randomized, double-blind, placebo-controlled crossover design increased the reliability of the study by reducing interindividual variability and allowing each participant to act as their own control.

Despite these strengths, some limitations should be noted. Only a single dose of 100 mg was administered in the study, limiting conclusions about the long-term bioavailability and cumulative effects of CoQ-10 supplementation. Although the 24-h blood sampling captured short-term pharmacokinetics, CoQ-10 has a long plasma half-life (~33 h) (5), and a longer observation period could have provided additional insight into its elimination kinetics. The sample size (*n* = 18; 7 males, 11 females) did not allow the analysis of potential gender differences in CoQ-10 uptake. A previous study reported that women had higher plasma CoQ-10 concentrations than men 48 h after ingestion. This suggests that gender-specific factors may influence CoQ-10 metabolism and clearance rates (26). Although standardized meals were administered in the study, the effects of different fat compositions (e.g., high-fat vs. low-fat meals) on CoQ-10 absorption were not examined, which may have influenced the bioavailability results due to its fat-soluble nature. Future studies should examine these variables by including larger cohorts, gender-specific analyses, and longer supplementation periods to evaluate sustained effects on oxidative stress, inflammation, and lipid metabolism.

While the present trial focused solely on pharmacokinetic outcomes, the enhanced absorption observed with the liposomal formulation may have implications for clinical efficacy in conditions where CoQ-10 supplementation is beneficial. The significantly higher bioavailability of liposomal CoQ-10, as evidenced by a 31.3% higher Cmax and a 22.6% higher AUC0-24 compared with standard CoQ-10, suggests that liposomal formulations are a superior alternative for individuals who require higher plasma levels of CoQ-10 without increasing dosage. This is particularly beneficial for people with gastrointestinal disease, pancreatic insufficiency, or bile acid deficiency, where lipid metabolism is impaired, making absorption of standard CoQ-10 inefficient. In clinical practice, liposomal CoQ-10 may be a more effective therapeutic option for cardiovascular patients and the elderly who require a continuous supply of CoQ-10 to support mitochondrial function, reduce oxidative stress, and improve endothelial cell health. From a sports nutrition perspective, the enhanced absorption profile could lead to better ATP production, faster muscle recovery, and improved antioxidant protection, making it an optimal choice for endurance and strength athletes. Future studies should evaluate whether these pharmacokinetic improvements translate into measurable benefits in various populations, including those with cardiovascular, neurological, or metabolic disorders. Such research could also explore the effects of chronic supplementation, varying dosages, and different timing of administration relative to meals.

## 5 Conclusion

Liposomal delivery significantly enhances CoQ-10 absorption.

## Data Availability

The raw data supporting the conclusions of this article will be made available by the authors, without undue reservation.
